# Supplementary material for: A megaplasmid family driving dissemination of multidrug resistance in Pseudomonas
Source: Nat Commun. 2020 Mar 13;11:1370. doi: 10.1038/s41467-020-15081-7 (PMC7070040; doi:10.1038/s41467-020-15081-7)
Supplement: Supplementary file 1 — Supplementary information [file 41467_2020_15081_MOESM1_ESM.pdf]

Supplementary Information

A megaplasmid family driving dissemination of multidrug resistance in *Pseudomonas*

Cazares et al.

Supplementary Fig. 1. Thai megaplasmids maps and pairwise comparison

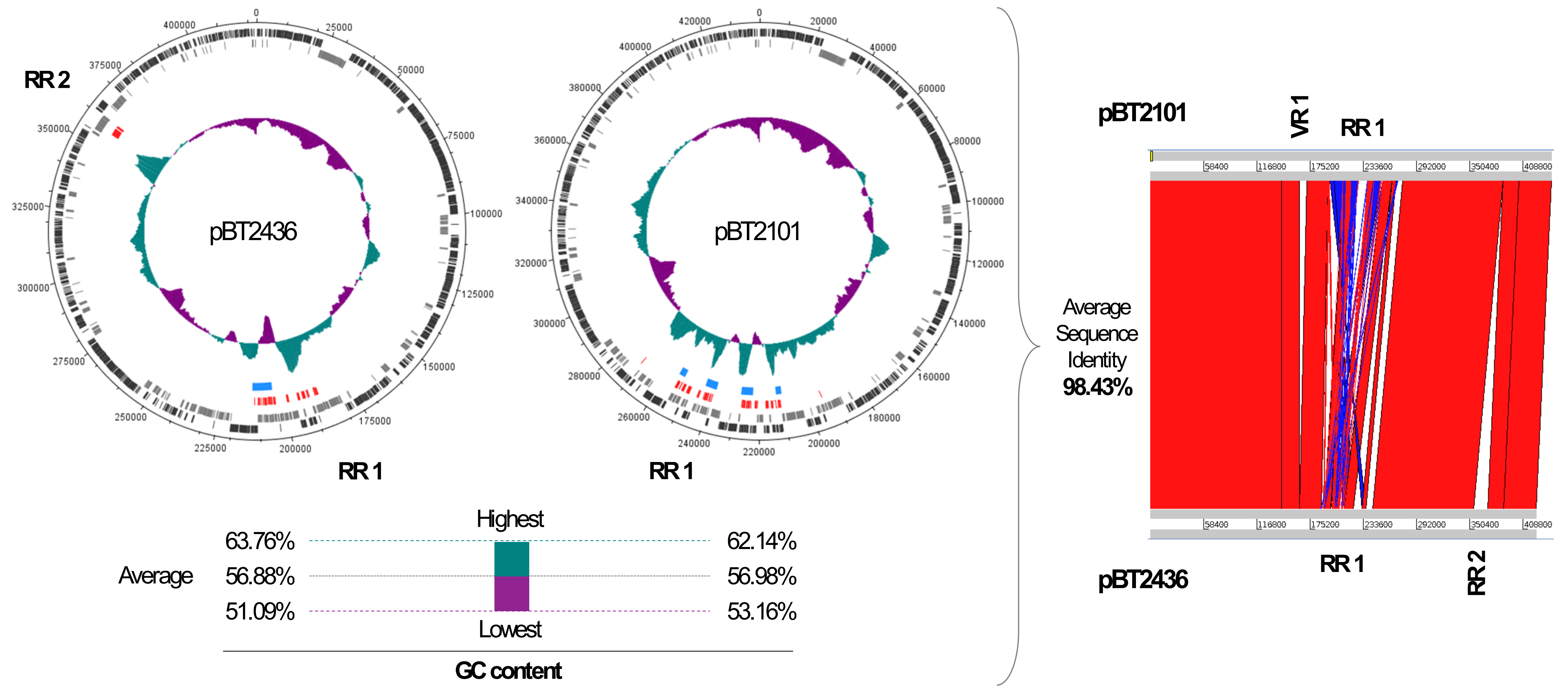

Left: pBT2436 and pBT2401 maps showcasing the location of AMR genes (red blocks) and genes encoding transposases or integrases (blue blocks). Innermost circle shows the GC content distribution of the megaplasmid genomes whereas the two outermost grey rings represent the ORFs encoded in the negative and positive strands. Right: Pairwise comparison of the pBT2436 and pBT2101 nucleotide sequences. Location of the Resistance (RR) and Variable (VR) regions of the megaplasmids is indicated.

Supplementary Fig. 2. Duplications detected in pBT2436 and pBT2101 AMR regions

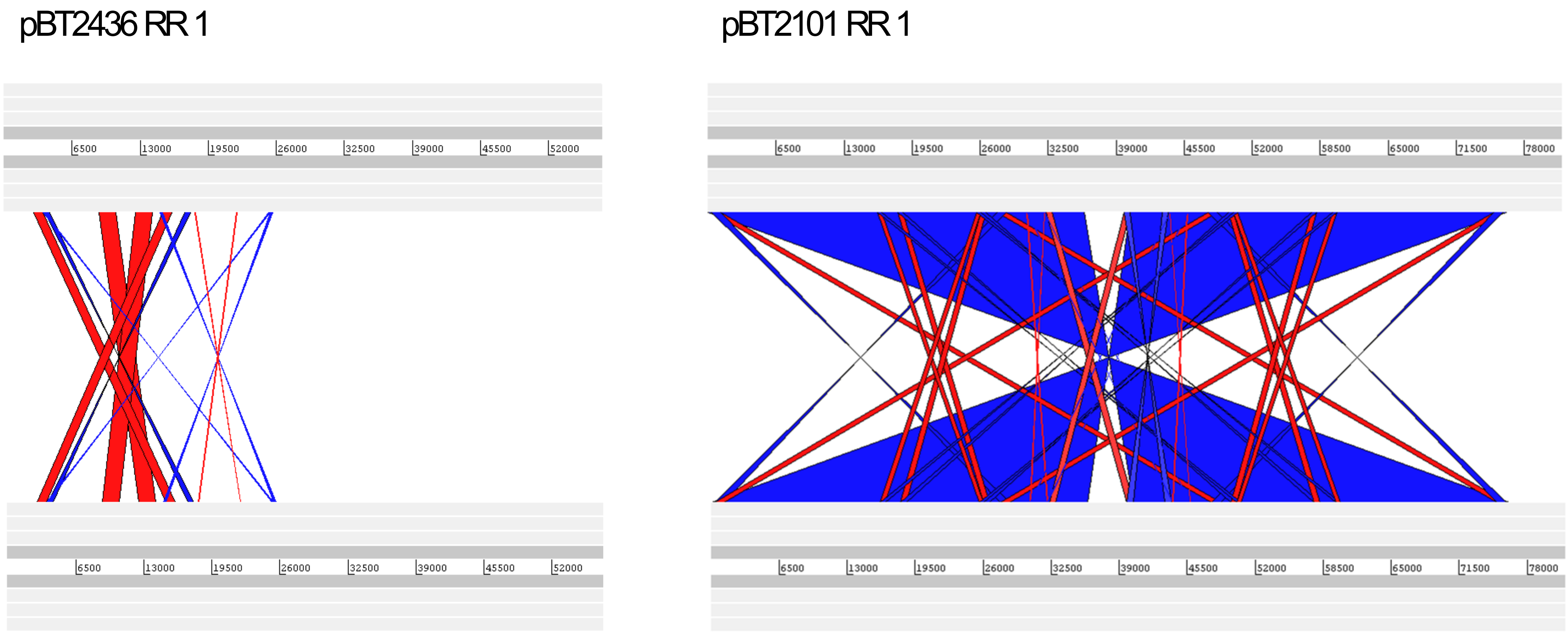

Self-comparison of RR1 regions of the indicated megaplasmids at nucleotide level. Colour blocks connecting the AMR regions represent matches featuring >97% sequence identity in either the forward (red) or reverse complement (blue) direction. Overall self-similarity between the compared regions was masked to visualize discrete repeated regions. Note that the coordinates indicated correspond to the RR1 regions and not to the whole megaplasmid sequences.

Supplementary Fig. 3. Comparative analysis of the pBT2436 Resistance Region 2

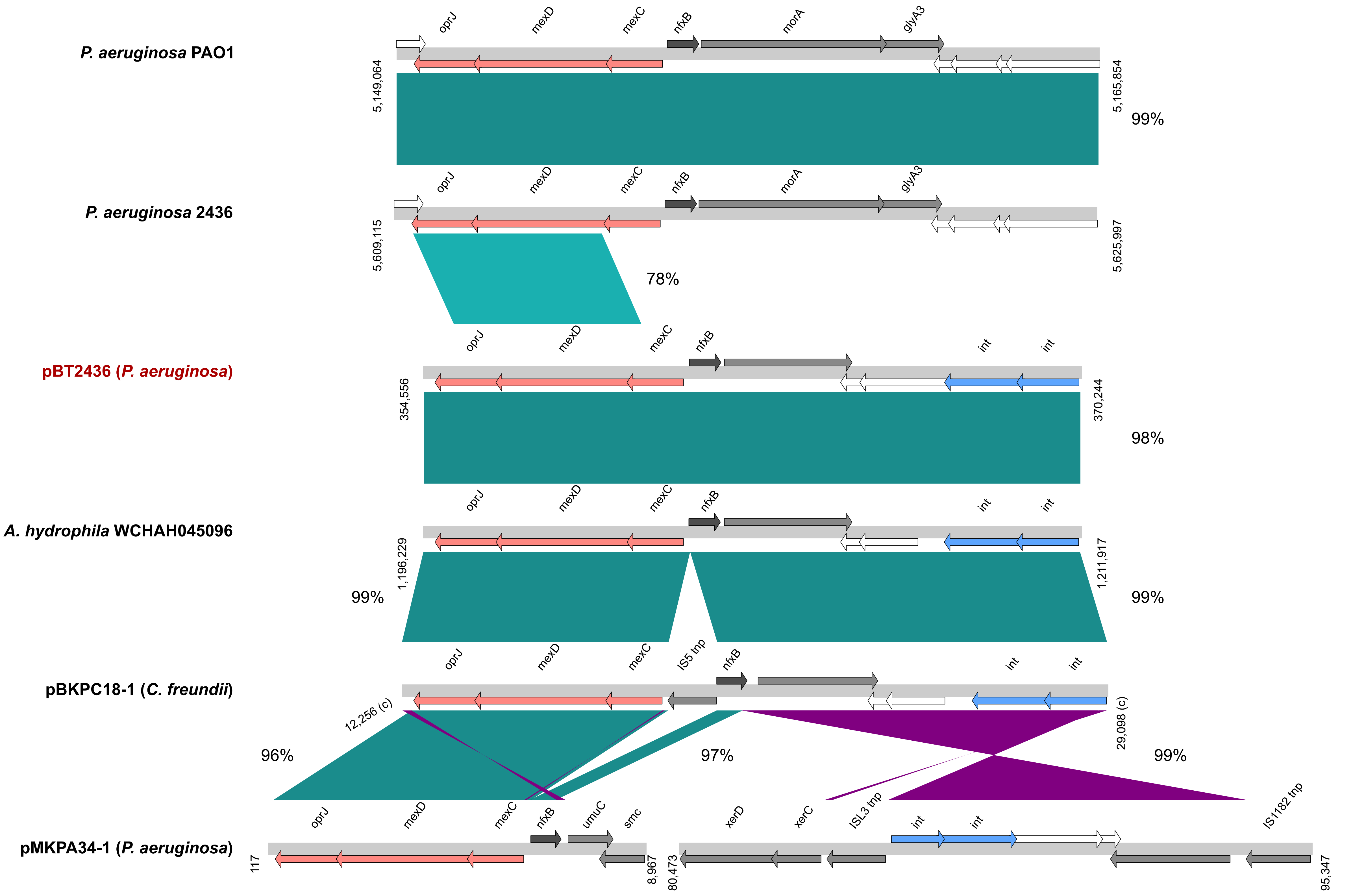

The figure shows the pairwise comparisons at nucleotide level of sequences homologous to the pBT2436 RR2 (labeled in red). Compared regions correspond to chromosomal segments of the *P. aeruginosa* strains PAO1 and 2436 (pBT2436 carrier), the *A. hydrophila* strain WCHAH045096, and regions from the plasmids pBKPC18-1 and pMKPA34-1 of *C. freundii* and *P. aeruginosa*, respectively. The percentage of sequence identity detected in the homologous regions, depicted as green (forward) or magenta (reverse complement) connecting blocks, is indicated next to the corresponding matches. Coordinates of the compared regions in their corresponding genomes are shown flanking the maps, (c) denotes the sequence was changed to the reverse complement direction to ease the comparison visualization. ORFs in the regions are depicted as arrows and their colour code denotes: genes encoding the structural components of the MexCD-OprJ efflux pump (red) and its regulator NfxB (dark grey), genes encoding integrases (blue), and other genes with known (light grey) or unknown (white) function. Names of genes of interest are indicated above the corresponding arrows.

Supplementary Fig. 4. Kmer-based tree of *P. aeruginosa* genomes from Thailand

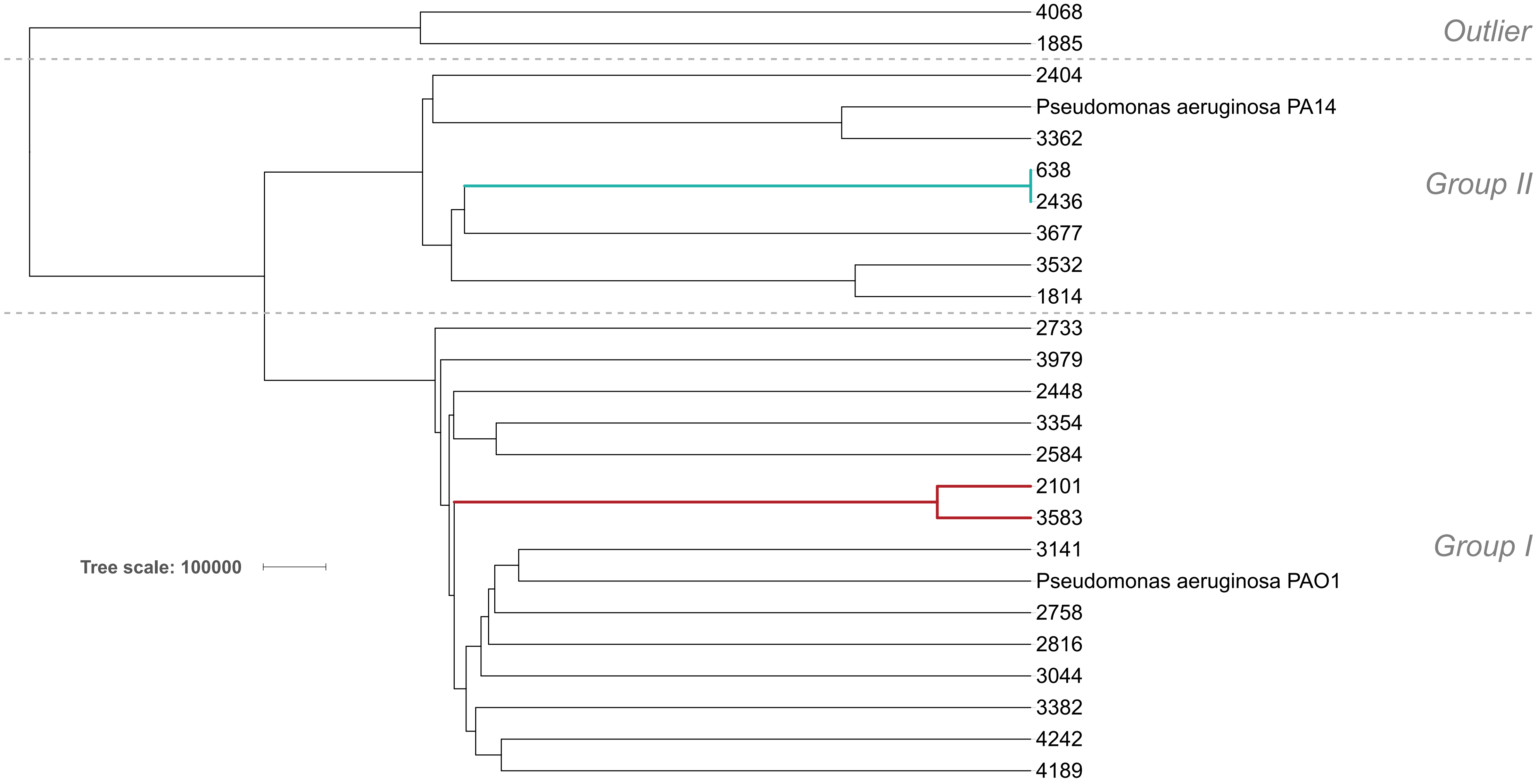

The tree shows the relationship existing among the 23 *P. aeruginosa*Thai clinical isolates from this study and two reference strains (PAO1 and PA14) based on k-mer sequence clustering. Note that the resulting phylogeny reflects the structure of the wider *P. aeruginosa* population (Groups I and II are indicated). Isolates identified as pBT2436-like megaplasmid carriers are indicated in cyan and red branches.

Supplementary Fig. 5. Identification of pBT2436-like megaplasmids from short-read sequencing data of Thai *P. aeruginosa* clinical isolates

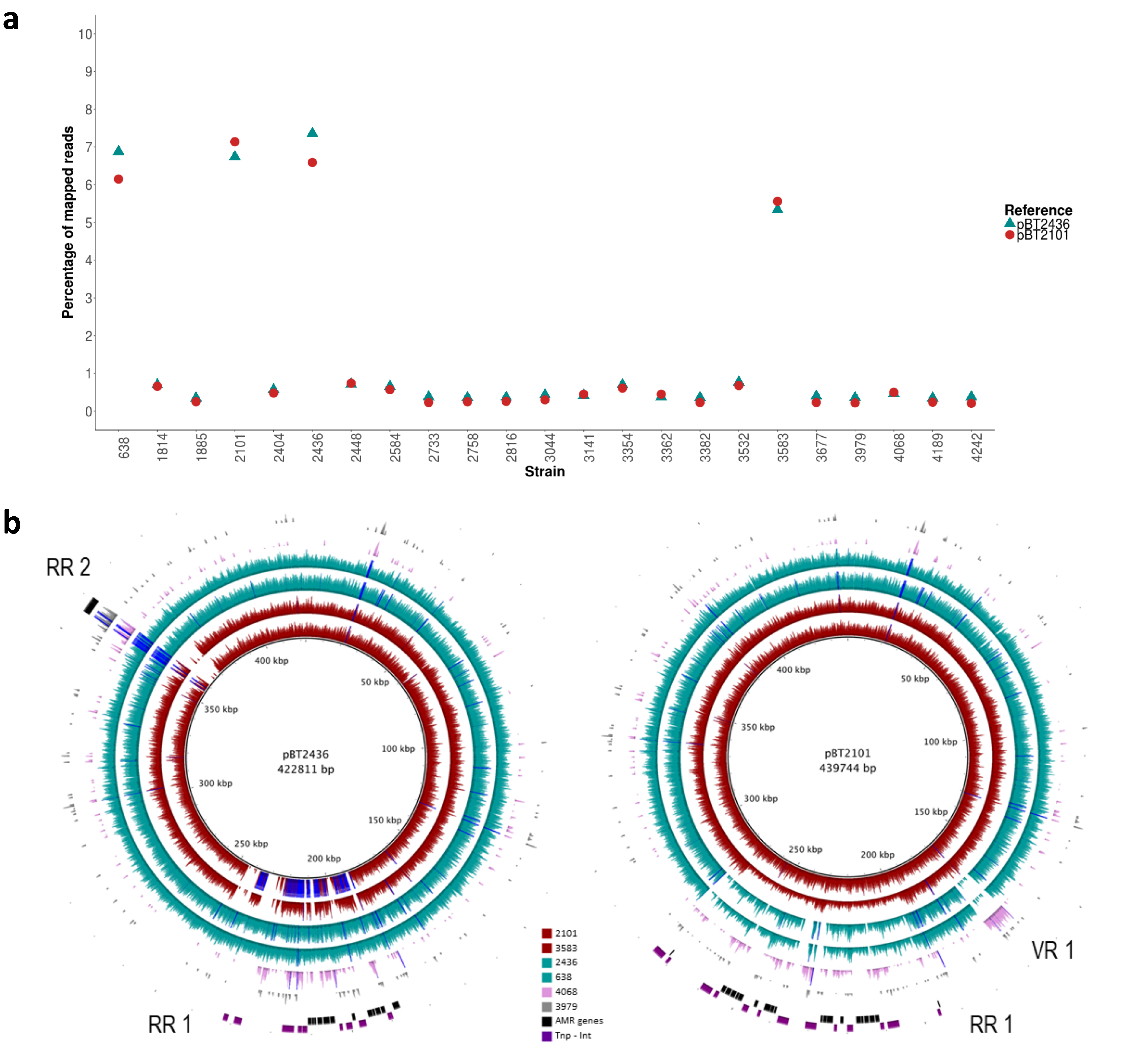

The percentage of sequencing reads of the analyzed genomes mapping pBT2436 (green triangles) or pBT2101 (red dots) is plotted in **a**. Note that genomes of the strains 2436/2101 and 4068 represent positive and negative controls, respectively, as the presence/absence of pBT436-like megaplasmids in them was previously determined by long-read sequencing. The visualization of the reads alignment against the reference genomes for selected strains is shown in **b**. The first four rings (from the innermost to outermost) correspond to strains displaying the highest percentage of mapped reads whereas the next two rings represent the distribution of mapped reads of the negative control (4068, pink) and a strain categorized as lacking pBT2436-like megaplasmids (3979, grey). Blue regions in the rings denote mapping coverage values above the threshold and feature around twice the number of mapped reads than in the rest of the genome. Location of AMR genes, and those encoding integrases or transposases in the reference genomes, is shown in the two outermost black and purple rings, respectively. Resistance (RR) and variable (VR) regions in pBT2436 and pBT2101 are also indicated.

Supplementary Fig. 6. Pangenome analysis of the pBT2436-like megaplasmid family

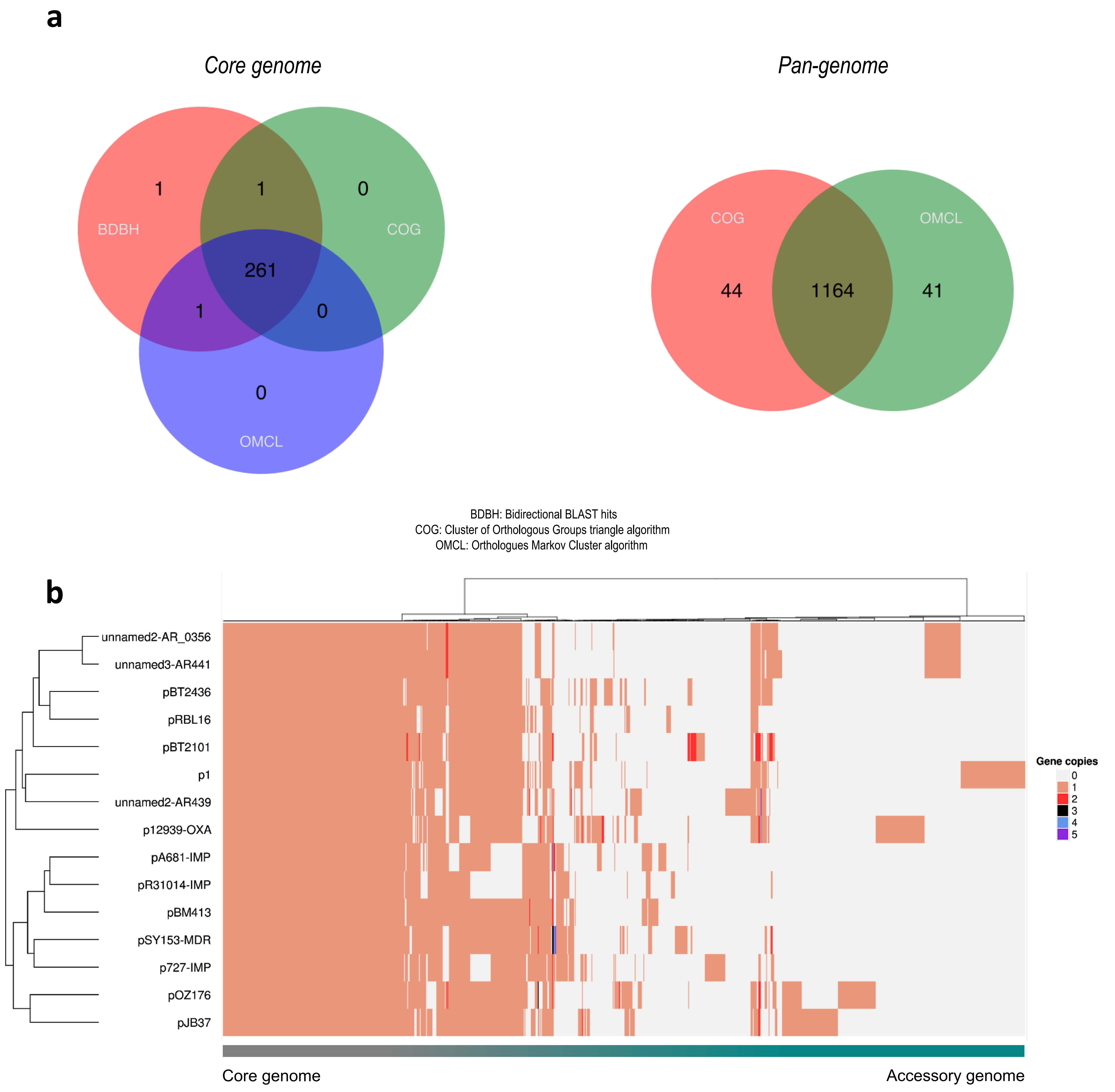

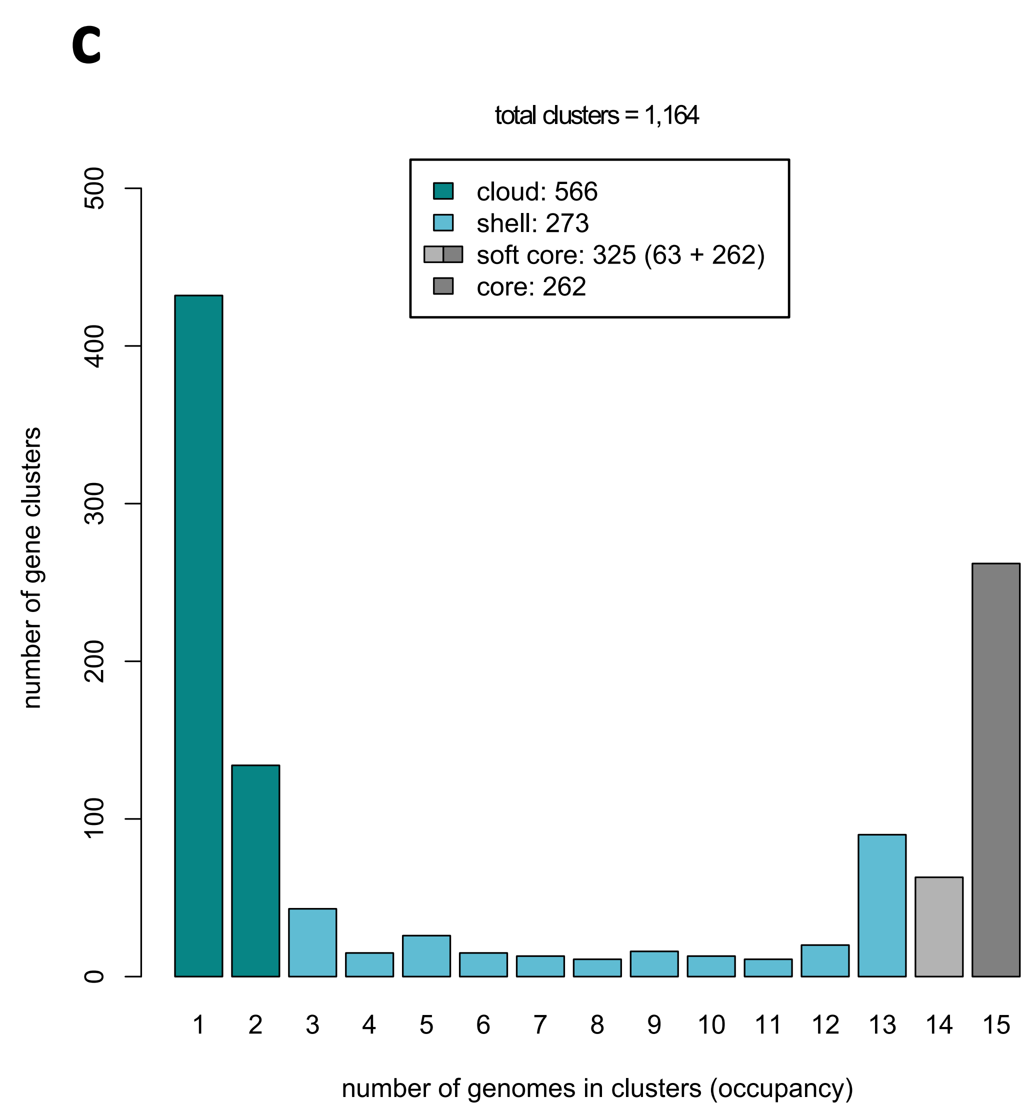

d

Plasmid	Soft-core	Shell	Cloud	Unique
p1	324	168	98	93
p12939-OXA	308	169	96	75
p727-IMP	322	149	36	30
pA681-IMP	311	127	22	11
pBM413	324	185	11	9
pBT2101	324	161	22	18
pBT2436	324	186	17	12
pJB37	323	165	90	52
pOZ176	317	169	103	57
pR31014-IMP	323	97	9	6
pRBL16	323	151	9	7
pSY153-MDR	323	183	25	19
unnamed2-AR439	316	159	50	37
unnamed2-AR_0356	325	164	54	1
unnamed3-AR441	325	160	58	5
	Average			
	320.8	159.5	46.7	28.8

(a) Venn diagrams highlighting the intersection between the number of gene families computed by different clustering algorithms to identify the core (left) and pangenome of the pBT2436-like megaplasmid group. BDBH: Bidirectional BLAST hits, COG: Cluster of Orthologous Groups triangle algorithm, OMCL: Orthologues Markov Cluster algorithm. **(b)** Pangenome matrix of the pBT2436-like megaplasmids family. The heatmap represents the gene content, in terms of presence/absence, of the pBT2436-like megaplasmids pangenome inferred from the comparison of fifteen members with complete genomes. Comparison was performed at protein level rendering 1164 consensus groups as the result of the intersection between the COG- and OMCL-based clustering strategies. Protein groups (X axis) and megaplasmids (Y axis) are hierarchically clustered based on the "ward.D" and "complete" methods, respectively, with Euclidean distance. Number of gene copies per cluster/per genome are colour-coded and numbered from 0 (grey:absence) to 5. Distribution of the pangenome in the core and accessory components is indicated. **(c)** Pangenome structure analysis displaying the classification of the pangenome consensus clusters into cloud, shell, soft-core and core compartments. **(d)** Breaking-up of the number of sequence clusters identified per genome/pangenome compartment.

Supplementary Fig. 7. Functional annotation of the pBT2436-like megaplasmids family accessory genome

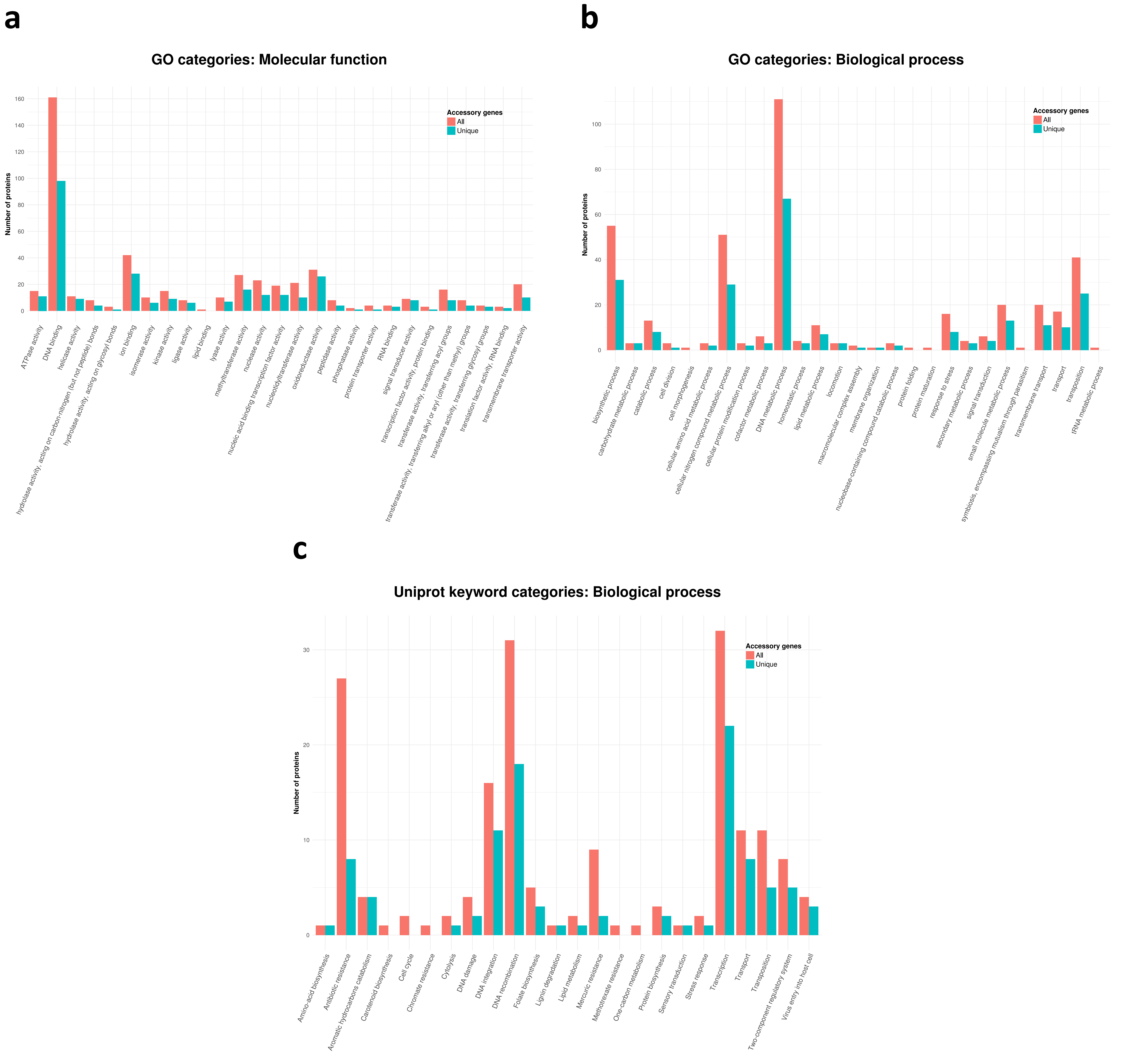

Protein annotation was performed with the Sma3s tool and functional classification is based on the identified GO-terms (**a** and **b**) and Uniprot keywords (**c**) (Supplementary Data 3). Plots **a** and **b** show the number of accessory proteins classified into different "Molecular Function" and "Biological Process" GO-terms categories, respectively. The three plots compare the classification of all the accessory proteins of the megaplasmids group regarding those that were identified as unique in the pangenome, i.e. plasmid-specific proteins.

Supplementary Fig. 8. Multiple sequence alignment of RepA proteins from complete megaplasmids

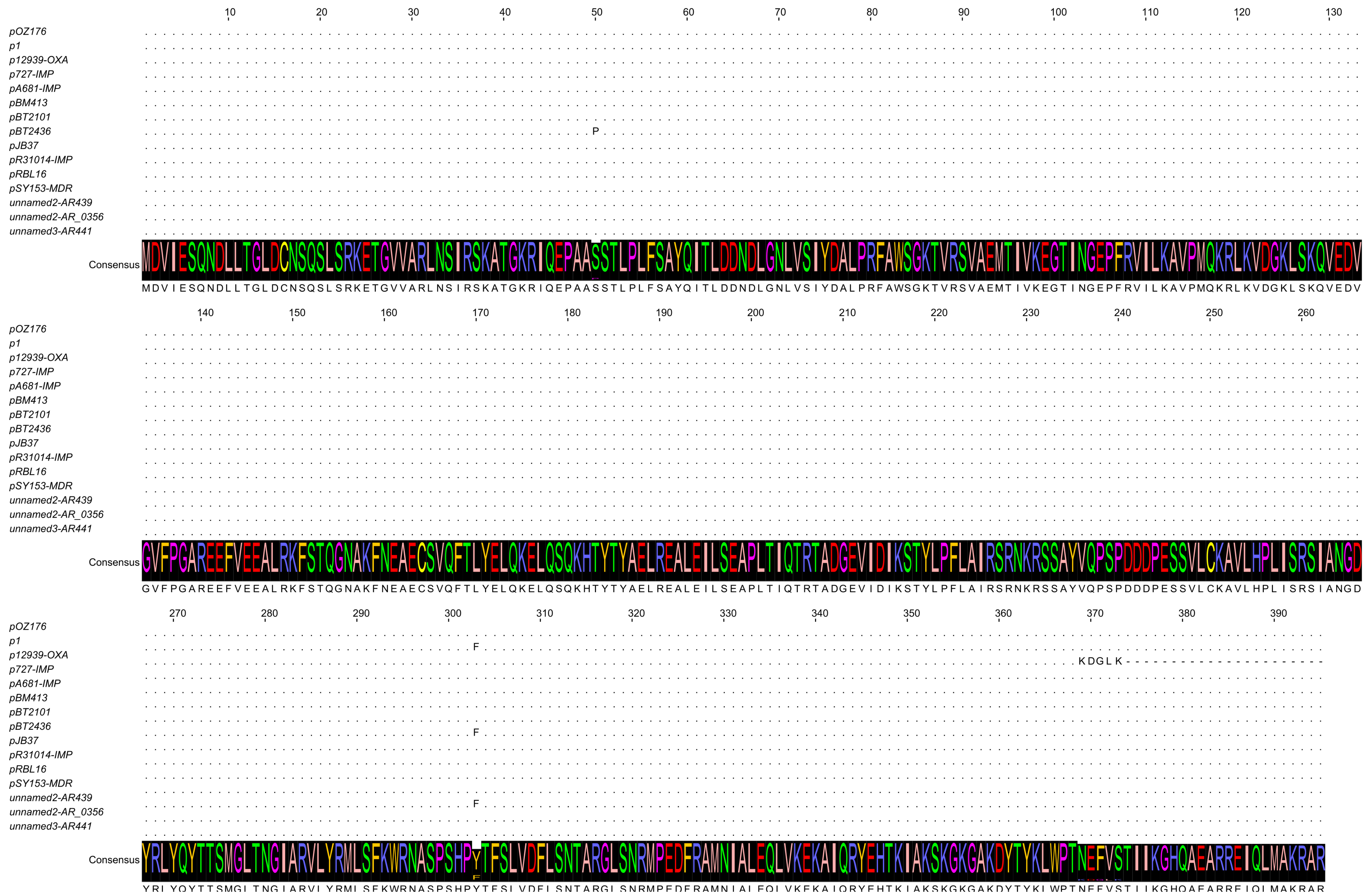

Identical amino acids in the alignment are represented as dots to highlight the few differences identified between the protein sequences compared. Coordinates are included at the top of the alignment as reference.

Supplementary Fig. 9. Search of pBT2436-like megaplasmids in *Pseudomonas* genomes from the GenBank Assembly database

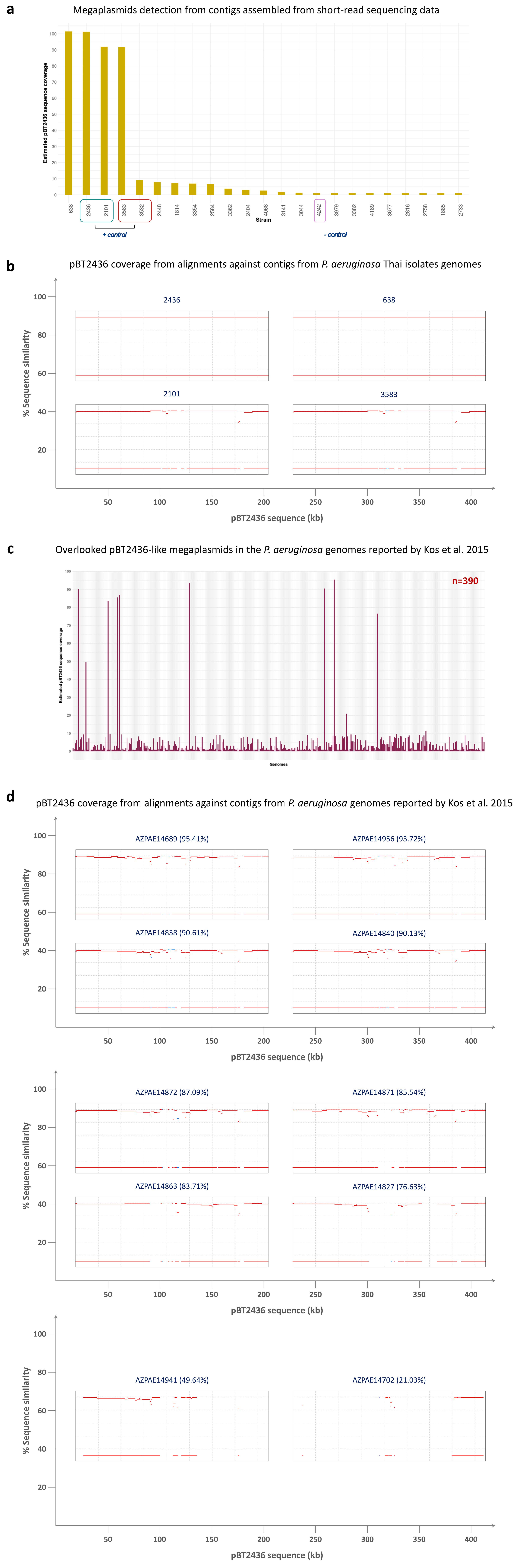

Presence of pBT2436-like megaplasmids was assessed by aligning the query genomes to the pBT2436 nucleotide sequence and estimating its coverage from the alignments. Graph in **a** correspond to our pilot test and shows the pBT2436 coverage estimated from the alignments against a set of Illumina-generated genome assemblies of *P. aeruginosa* clinical isolates from Thailand. Positive and negative controls, where the presence or absence of pBT2436-related megaplasmids was previously determined by long-read sequencing and/or mapping of short-sequencing reads, are indicated. A visualization of the pBT2436 coverage (X axis) from the top-4 alignments, and their corresponding percentage of similarity (Y axis) is shown in **b**. Plots in **c** and **d** display similar data to **a** and **b** for the analysis of the 390 *P. aeruginosa* genomes reported by Kos et al. 2015.

Supplementary Fig. 10. MLST-based Sequence Types (STs) of *P. aeruginosa* genomes identified as megaplasmid carriers

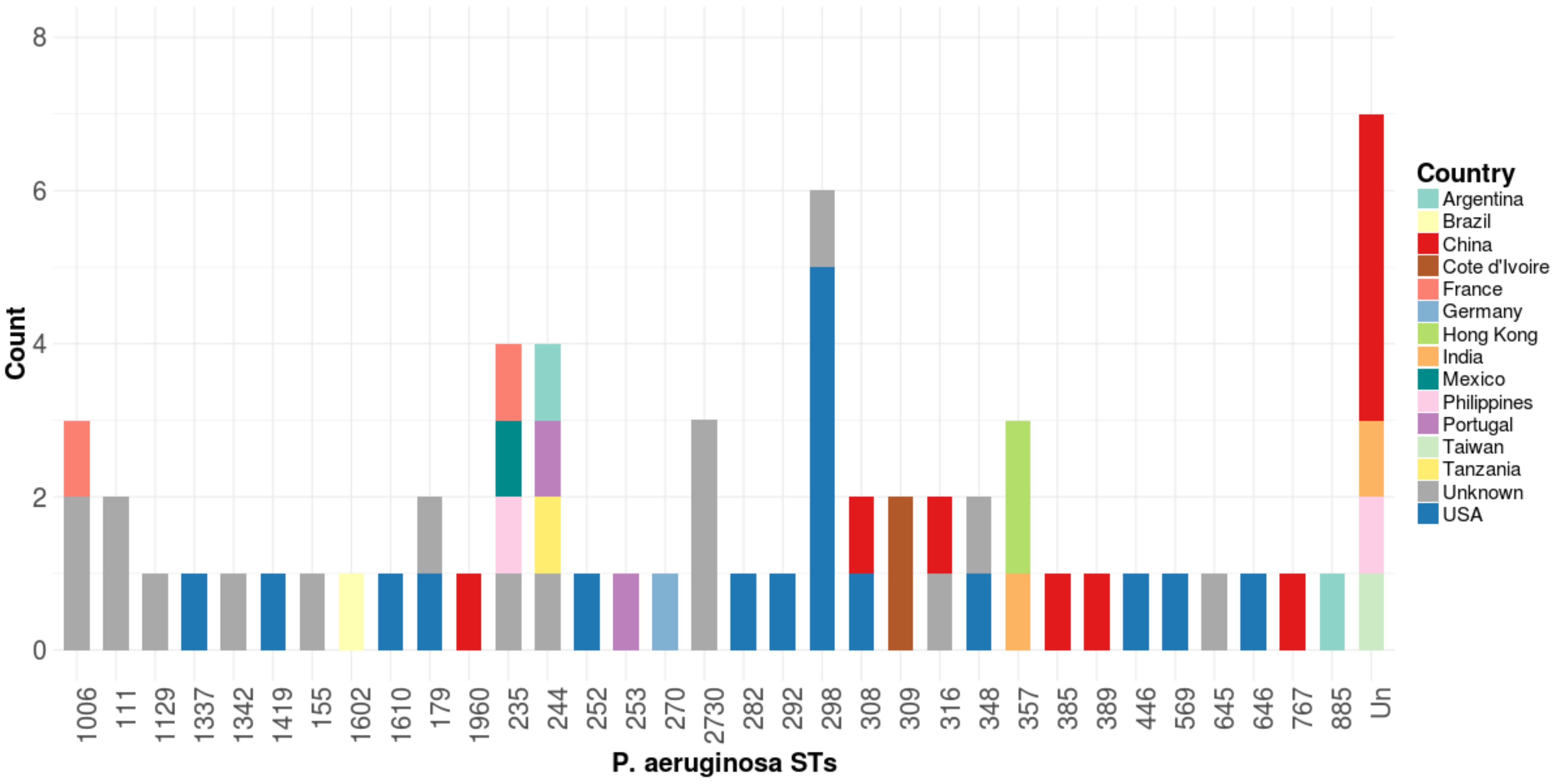

Plotted are the number of megaplasmid-carrier genomes from GenBank belonging to 33 different *P. aeruginosa* ST. “Un” stand for unidentified ST. The country of isolation of the samples is indicated.

Supplementary Fig. 11 pBT2436-like megaplasmids are transfer competent

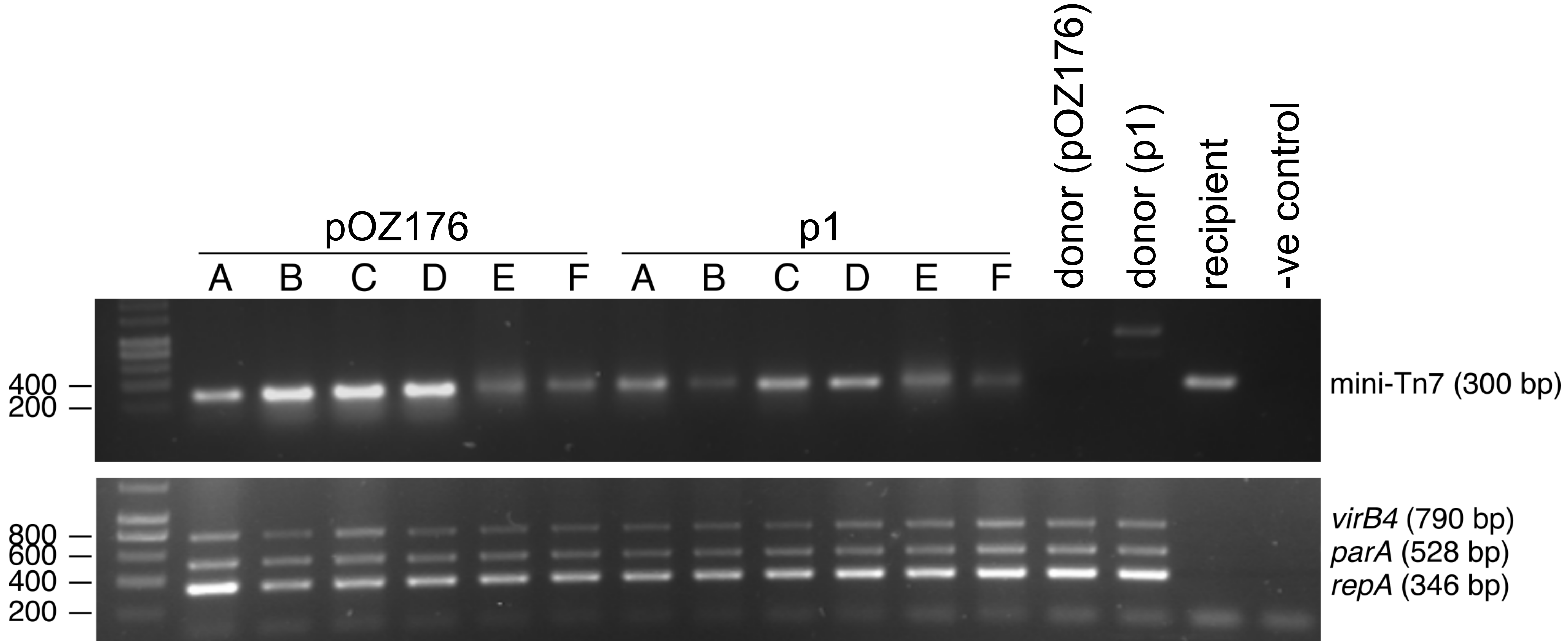

Colony PCR analysis of *P. fluorescens* SBW25 transconjugants that have acquired pOZ176 or p1. Primer sequences are provided in Supplementary Table 2 and reaction conditions are described in Methods. Source data are provided as a Source Data file.

Supplementary Table 1. Extended antibiotic susceptibility profile of four multidrug resistant Thai clinical isolates

Strain	Penicillins				Cephalosporins			Carbapenems			Monobactams	Fluoroquinolones		Aminoglycosides			
	**Pip**	**Pip/T**	**Tic**	**Tic/C**	**Cefep**	**Ceftaz**	**Cef/A**	**Dori**	**Imi**	**Mer**	**Azt**	**Cip**	**Lev**	**Ami**	**Gen**	**Net**	**Tob**
2436	R	R (15)	R	R	R	R	R	R	R	R	R	R	R	R (0)	R	R (0)	R
638	R	S (20)	R	R	R	R	R	R	R	R	R	R	R	R (0)	R	S (13)	R
2101	R	R (12.3)	R	R	R	R	R	R	R	R	R	R	R	S (22.3)	R	R (11)	R
3583	R	R (16)	R	R	R	R	R	R	R	R	R	R	R	S (22.3)	R	S (13)	R

Where variations between strains were observed, mean values (in mm) for zones of inhibition (from three replicates) are shown in brackets. Breakpoints, according to EUCAST guidelines, were as follow: 18 mm for Pip/T; 15-18 mm for Ami; 12 mm for Net; 16 mm for Tob.

Supplementary Table 2. Primers used in this study

Primer name	Sequence	Target (Locus tag) *	Product size (bp)	Purpose	Reference
repA_F	GATGTTGGTGTTTTCCCCGG	putative repA (PkP19E3_30450 / PA96_RS36150)	346	Detecting pBT2436-family megaplasmids	This work
repA_R	AACTCTCAGGGTCGTCATCC				
parA_F	ACTTCTCCAGTCGACTCGC	putative parA (PkP19E3_30450 / PA96_RS36150)	528	Detecting pBT2436-family megaplasmids	This work
parA_R	CGGTATCAATTGCACCTCGG				
virB4_F	ACCCCATCTGTATCAACCCG	putative virB4/traC (PkP19E3_32130 / PA96_RS34470)	790	Detecting pBT2436-family megaplasmids	This work
virB4_R	CAAAGCTCAATCTCCTCGGC				
Tn7-GlmS-pf1F	CGTCATCAACATGCCGCACATC	boundary of mini-Tn7 insertion site in *P. fluorescens* SBW25	300	Detecting labelled *P. fluorescens* SBW25	Koch et al. 2001
Tn7R109	CAGCATAACTGGACTGATTTCAG				
P19E3_chr_virB4_F	CAGGCAATCACTTCGAGAGC	*P. koreensis* P19E3 putative chromosomal virB4 (PkP19E3_29665)	198	Detecting *P. koreensis* P19E3 MGEs	This work
P19E3_chr_virB4_R	AGTCAATTTCCGGCATCACG				
P19E3_p2_parA_F	GGGATTTCTGGTGATCGTGC	*P. koreensis* P19E3 putative plasmid p2 parA (PkP19E3_32850)	333	Detecting *P. koreensis* P19E3 MGEs	This work
P19E3_p2_parA_R	GAGAAAATCGCTGGCAACCA				
P19E3_p3_rep_F	AAGACAACCAAACCATCCGC	*P. koreensis* P19E3 putative plasmid p3 repA (PkP19E3_34395)	541	Detecting *P. koreensis* P19E3 MGEs	This work
P19E3_p3_rep_R	TTCTTCATCTGCGACTCGGT				

* PkP19E3 Locus tags correspond to the p1 sequence

Supplementary References

Koch B., Jensen L. E. & Nybroe O. A panel of Tn7-based vectors for insertion of the gfp marker gene or for delivery of cloned DNA into Gram-negative bacteria at a neutral chromosomal site *J Microbiol Methods* 45 187–195 10.1016/S0167-7012(01)00246-9 (2001).

Kos, V. N. et al. The resistome of Pseudomonas aeruginosa in relationship to phenotypic susceptibility. Antimicrob. Agents Chemother. 59, 427–436 (2015).
