# Supplementary material for: A megaplasmid family driving dissemination of multidrug resistance in Pseudomonas
Source: Nat Commun. 2020 Mar 13;11:1370. doi: 10.1038/s41467-020-15081-7 (PMC7070040; doi:10.1038/s41467-020-15081-7)
Supplement: Supplementary file 3 — Description of Additional Supplementary Files [file 41467_2020_15081_MOESM3_ESM.pdf]

Description of Additional Supplementary Files

File Name: Supplementary Data 1
Description: General features of the Thailand clinical isolates and their genome sequences

File Name: Supplementary Data 2
Description: pBT2436 and pBT2101 genome annotation

File Name: Supplementary Data 3
Description: Functional annotation of the pBT2436-like family accessory genome

File Name: Supplementary Data 4
Description: List of megaplasmid relatives identified in *Pseudomonas* genomes from the GenBank assembly database and their associated metadata
