# Supplementary material for: A megaplasmid family driving dissemination of multidrug resistance in Pseudomonas
Source: Nat Commun. 2020 Mar 13;11:1370. doi: 10.1038/s41467-020-15081-7 (PMC7070040; doi:10.1038/s41467-020-15081-7)
Supplement: Supplementary file 8 — Reporting Summary [file 41467_2020_15081_MOESM8_ESM.pdf]

nature research

Corresponding author(s): Adrian Cazares and Craig Winstanley

Last updated by author(s): Feb 5, 2020

# Reporting Summary

Nature Research wishes to improve the reproducibility of the work that we publish. This form provides structure for consistency and transparency in reporting. For further information on Nature Research policies, seeAuthors & Referees and theEditorial Policy Checklist .

## Statistics

For all statistical analyses, confirm that the following items are present in the figure legend, table legend, main text, or Methods section.

| n/a | Confirmed | |
|---|---|---|
| ☐ | ☒ | The exact sample size (*n*) for each experimental group/condition, given as a discrete number and unit of measurement |
| ☐ | ☒ | A statement on whether measurements were taken from distinct samples or whether the same sample was measured repeatedly |
| ☐ | ☒ | The statistical test(s) used AND whether they are one- or two-sided<br>*Only common tests should be described solely by name; describe more complex techniques in the Methods section.* |
| ☒ | ☐ | A description of all covariates tested |
| ☒ | ☐ | A description of any assumptions or corrections, such as tests of normality and adjustment for multiple comparisons |
| ☐ | ☒ | A full description of the statistical parameters including central tendency (e.g. means) or other basic estimates (e.g. regression coefficient) AND variation (e.g. standard deviation) or associated estimates of uncertainty (e.g. confidence intervals) |
| ☐ | ☒ | For null hypothesis testing, the test statistic (e.g. *F*, *t*, *r*) with confidence intervals, effect sizes, degrees of freedom and *P* value noted<br>*Give P values as exact values whenever suitable.* |
| ☒ | ☐ | For Bayesian analysis, information on the choice of priors and Markov chain Monte Carlo settings |
| ☒ | ☐ | For hierarchical and complex designs, identification of the appropriate level for tests and full reporting of outcomes |
| ☒ | ☐ | Estimates of effect sizes (e.g. Cohen's *d*, Pearson's *r*), indicating how they were calculated |

*Our web collection on statistics for biologists contains articles on many of the points above.*

## Software and code

Policy information about availability of computer code

| | |
|---|---|
| Data collection | Data collection was carried out using sequencing with commercial providers and by accessing public genome databases as described in the methods. Accession numbers for all the analised data is provided. |
| Data analysis | The following software was used to analyse the data presented in this study:<br>* Genome Assembly:<br>HGAP v.3 workflow from SMRT Portal v.2.3.0<br>Circlator v.1.5.3<br>PILON v.1.22<br>Cutadapt v.1.2.1<br>Sickle v.1.200<br>A5 MiSeq assembly pipeline<br>QUAST v.3.1<br>* Annotation:<br>MLST tool v.2.8<br>ABRicate tool v.0.8<br>Prokka v.1.12<br>InterProScan v.5.30-69.0<br>Sma3s v.2<br>Integron Finder v.1.5.1<br>ISfinder<br>* Comparative / Phylogenomics analysis:<br>blast+ v2.5.0+<br>Artemis Comparison Tool (ACT) v.17.0.1 |

BRIG v.0.95
GET_HOMOLOGUES v.10092018
GGET_PHYLOMARKERS v.2.2.5
IQ-tree v1.5.5
iTOL v.4.3.3
MUMmer v.3.1
SaffronTree v.0.1.2
* Genome mapping:
BWA-MEM v.0.7.17-r1188
Samtools v.1.7
* Other data analysis/visualization:
ComplexHeatmap package v.1.17.1

When not using the default settings reported for the used programs, modifications are stated in the methods.

For manuscripts utilizing custom algorithms or software that are central to the research but not yet described in published literature, software must be made available to editors/reviewers. We strongly encourage code deposition in a community repository (e.g. GitHub). See the Nature Research guidelines for submitting code & software for further information.

## Data

Policy information about availability of data

All manuscripts must include a data availability statement. This statement should provide the following information, where applicable:

- Accession codes, unique identifiers, or web links for publicly available datasets
- A list of figures that have associated raw data
- A description of any restrictions on data availability

The genome sequence data generated in this study have been deposited in GenBank under the BioProjects PRJNA540594 (PacBio) and PRJNA325248 (Illumina). The BioSample and Sequence accession numbers of every strain sequenced in this work are listed in the Supplementary Data 1. The accession numbers corresponding to the analised genomes from GenBank that we identified as megaplasmid-carriers are listed in the Supplementary Data 4.

Additionally, the information of the megaplasmids annotation, including their protein sequences, is provided in the Supplementary Data files 2 and 3.

# Field-specific reporting

Please select the one below that is the best fit for your research. If you are not sure, read the appropriate sections before making your selection.

- [x] Life sciences
- [ ] Behavioural & social sciences
- [ ] Ecological, evolutionary & environmental sciences

For a reference copy of the document with all sections, see nature.com/documents/nr-reporting-summary-flat.pdf

# Life sciences study design

All studies must disclose on these points even when the disclosure is negative.

**Sample size**

The bacterial strains genome sequenced in this study correspond to Pseudomonas aeruginosa clinical isolates acquired from patients with diverse type of infections in the Ramathibodi Hospital, Mahidol University, Bangkok, Thailand.

The existing genome data analised in this sudy correspond to various strains of the Pseudomonas genus from diverse isolation origins and is publicly available. Accession numbers to this data are provided in the Supplementary Data 4.

No sample size calculation was carried out. Pseudomonas aeruginosa strains were isolated from a variety of biological samples aiming to represent a variety of infection types. Further strain selection for genome sequencing aimed to represent the diversity of antibiotic-resistance profiles observed during the bacterial strain characterization.

For the analaysis of the existing genome data, we focused on the Pseudomonas genus, and further comparative investigation was performed only in datasets identified as megaplasmid carriers.

**Data exclusions**

No data exclusion was applied to the initial analysis (genome sequencing and Pseudomonas genome data collection from public repositories). Further comparative analysis was performed only in sequence data displaying similarity to the pBT2436 megaplasmid nucleotide sequence.

**Replication**

The findings of this study are mostly supported by genome comparative analyses of both newly-generated and existing genome data. Detailed description of the software used to perform the bioinformatics analyses, data collection date, and accession to all the genome data analised are provided to ensure the reproducibility of our study.
The antibiotic susceptibility testing and guidelines used for its interpretations are also described in the methods. Plasmid stability assays were performed through a screening of 100 colonies and plasmid maintenance was confirmed in 20. Competition assays were performed with three independent transconjugant strains to control for any stochastic effects occurring during strain generation and four replicate competition assays were conducted for each strain.

**Randomization**

Randomization is not relevant to this study as no groups are considered.

| Blinding | This study is based on presence/absence, therefore, blinding was not relevant. |
| --- | --- |

# Reporting for specific materials, systems and methods

We require information from authors about some types of materials, experimental systems and methods used in many studies. Here, indicate whether each material, system or method listed is relevant to your study. If you are not sure if a list item applies to your research, read the appropriate section before selecting a response.

## Materials & experimental systems

| n/a | Involved in the study |
| --- | --- |
| ☒ | ☐ Antibodies |
| ☒ | ☐ Eukaryotic cell lines |
| ☒ | ☐ Palaeontology |
| ☒ | ☐ Animals and other organisms |
| ☒ | ☐ Human research participants |
| ☒ | ☐ Clinical data |

## Methods

| n/a | Involved in the study |
| --- | --- |
| ☒ | ☐ ChIP-seq |
| ☒ | ☐ Flow cytometry |
| ☒ | ☐ MRI-based neuroimaging |
